# Smartphone and Wearable Sensors for the Estimation of Facioscapulohumeral Muscular Dystrophy Disease Severity: Cross-sectional Study

**DOI:** 10.2196/41178

**Published:** 2023-03-15

**Authors:** Ahnjili Zhuparris, Ghobad Maleki, Ingrid Koopmans, Robert J Doll, Nicoline Voet, Wessel Kraaij, Adam Cohen, Emilie van Brummelen, Joris H De Maeyer, Geert Jan Groeneveld

**Affiliations:** 1 Centre for Human Drug Research (CHDR) Leiden Netherlands; 2 Department of Rehabilitation Rehabilitation Center Klimmendaal Nijmegen Netherlands; 3 Leiden Institute of Advanced Computer Science Leiden University Leiden Netherlands; 4 Facio Therapies Leiden Netherlands

**Keywords:** facioscapulohumeral muscular dystrophy, FSHD, smartphone, wearables, machine learning, Time Up and Go, regression, mobile phone, neuromuscular disease, mHealth, mobile health

## Abstract

**Background:**

Facioscapulohumeral muscular dystrophy (FSHD) is a progressive neuromuscular disease. Its slow and variable progression makes the development of new treatments highly dependent on validated biomarkers that can quantify disease progression and response to drug interventions.

**Objective:**

We aimed to build a tool that estimates FSHD clinical severity based on behavioral features captured using smartphone and remote sensor data. The adoption of remote monitoring tools, such as smartphones and wearables, would provide a novel opportunity for continuous, passive, and objective monitoring of FSHD symptom severity outside the clinic.

**Methods:**

In total, 38 genetically confirmed patients with FSHD were enrolled. The FSHD Clinical Score and the Timed Up and Go (TUG) test were used to assess FSHD symptom severity at days 0 and 42. Remote sensor data were collected using an Android smartphone, Withings Steel HR+, Body+, and BPM Connect+ for 6 continuous weeks. We created 2 single-task regression models that estimated the FSHD Clinical Score and TUG separately. Further, we built 1 multitask regression model that estimated the 2 clinical assessments simultaneously. Further, we assessed how an increasingly incremental time window affected the model performance. To do so, we trained the models on an incrementally increasing time window (from day 1 until day 14) and evaluated the predictions of the clinical severity on the remaining 4 weeks of data.

**Results:**

The single-task regression models achieved an *R*^2^ of 0.57 and 0.59 and a root-mean-square error (RMSE) of 2.09 and 1.66 when estimating FSHD Clinical Score and TUG, respectively. Time spent at a health-related location (such as a gym or hospital) and call duration were features that were predictive of both clinical assessments. The multitask model achieved an *R*^2^ of 0.66 and 0.81 and an RMSE of 1.97 and 1.61 for the FSHD Clinical Score and TUG, respectively, and therefore outperformed the single-task models in estimating clinical severity. The 3 most important features selected by the multitask model were light sleep duration, total steps per day, and mean steps per minute. Using an increasing time window (starting from day 1 to day 14) for the FSHD Clinical Score, TUG, and multitask estimation yielded an average *R*^2^ of 0.65, 0.79, and 0.76 and an average RMSE of 3.37, 2.05, and 4.37, respectively.

**Conclusions:**

We demonstrated that smartphone and remote sensor data could be used to estimate FSHD clinical severity and therefore complement the assessment of FSHD outside the clinic. In addition, our results illustrated that training the models on the first week of data allows for consistent and stable prediction of FSHD symptom severity. Longitudinal follow-up studies should be conducted to further validate the reliability and validity of the multitask model as a tool to monitor disease progression over a longer period.

**Trial Registration:**

ClinicalTrials.gov NCT04999735; https://www.clinicaltrials.gov/ct2/show/NCT04999735

## Introduction

Facioscapulohumeral muscular dystrophy (FSHD) is a progressive neuromuscular disease characterized by the wasting of muscles in the face, upper body, and legs [1]. The onset and progression vary greatly between individuals [2]. Early symptoms include difficulties in smiling, whistling, and shutting of the eyelids during sleep. These symptoms are followed by impaired upper-arm movements and walking. A total of 20% of individuals with FSHD eventually become wheelchair bound [2]. Less visible FSHD symptoms include fatigue and chronic pain [3]. In addition to the physical burden, individuals with FSHD also experience emotional, social, and socioeconomic burdens [4,5]. As a result, patients report increased deterioration in quality of life as the disease progresses [6].

Currently, there are no therapies or interventions that prevent the wasting of muscles in patients with FSHD [7]. Muscle-strengthening drugs have been shown to have limited effect on the disease progression [8]. As a result, patients with FSHD largely rely on symptomatic treatments (eg, analgesics, exercise, and cognitive therapy). The development of novel treatment options to delay or halt FSHD disease progression is currently under investigation [9,10]. However, measuring the effect of such new treatments is complicated, as disease progression is slow and no objective surrogate end points, predictive for clinical benefit, have been established.

Two common clinical assessments for assessing FSHD symptom severity are the FSHD Clinical Score and Timed Up and Go (TUG) test. The FSHD Clinical Score is composed of an evaluation of the extent of the muscle weakness among 6 regions of the body [11]. The TUG is a test used to assess functional mobility [12]. The test requires a participant to rise from a chair, walk 3 m forward, turn around, and return to the chair. These clinician-rate assessments provide a snapshot of the disease status and are primarily focused on muscular strength and function that are inherently subjective. Identifying novel objective biomarkers for monitoring disease progression could additionally provide clinically relevant insights and aid drug development. Novel digital end points for neuromuscular disease drug development have already demonstrated to be sensitive to differentiating patients from healthy volunteers and are strongly correlated with clinician assessments [13-15]. The widespread adoption of smartphones and wearables could provide new opportunities for objective and continuous monitoring of FSHD disease progression outside the laboratory.

This study was designed to identify smartphone-based and remote sensor–based features that could be used to assess FSHD disease severity. These features may enable the passive remote monitoring of disease progression and might potentially facilitate early detection of treatment effects on FSHD symptoms and the patient’s quality of life. We hypothesized that the behavioral features captured by these remote monitoring devices would capture the daily physical and social burden that patients with FSHD experience. Although other neuromuscular disease studies with similar protocols have used machine learning to construct their digital end points, until now, different monitoring periods were arbitrarily selected by various researchers [16,17]. Here, we investigated how different time windows affect the model’s performance to estimate one’s symptom severity over time [18,19]. As these features can vary considerably over time, we assessed the stability and test-retest reliability of the first week of data to estimate FSHD disease severity for the remainder of the trial. In this paper, we describe the development of a novel tool based on smartphone and remote sensor data to provide remote estimation of FSHD disease severity.

## Methods

### Overview

This study is an extension of a previous longitudinal clinical study that investigated the feasibility of monitoring and characterizing patients with FSHD and healthy controls in terms of biometric, physical, and social activities using data sourced from smartphones and other remote monitoring devices. Therefore, additional information regarding the data collection and data quality has been previously published [15].

### Patients

This was a noninterventional, cross-sectional study involving patients with FSHD. The study was performed between April and October 2019 in the Centre for Human Drug Research (CHDR) research unit in Leiden, the Netherlands. Table 1 provides an overview of the demographic distribution of the patients with FSHD enrolled in this study.

**Table 1:** An overview of characteristics of the FSHD^a^ participants (N=38).

Demographics		Values
**Gender, n**		
	Female	23
	Male	15
**Race, n**		
	African American or Black	—
	Mixed	1
	White	37
Age (years), mean (SD) (minimum, maximum)		44 (14.5) (18, 64)
Weight (kg), median (SD) (minimum, maximum)		79 (16) (52, 130)
BMI (kg/m^2^), median (SD) (minimum, maximum)		25 (4) (20, 44)
FSHD Clinical Score, median (SD) (minimum, maximum)		5 (3) (1, 13)
Timed Up and Go test (seconds), median (SD) (minimum, maximum)		7.7 (2.4) (5.5, 15.8)

^a^FSHD: facioscapulohumeral muscular dystrophy.

In total, 38 patients with genetically confirmed FSHD from the Netherlands and Belgium were included in the study. Eligible patients were 16 years or older, had genetically confirmed FSHD, and had an FSHD Clinical Score greater than zero. Patients had to be Android smartphone owners and willing to use either their own smartphone or an Android smartphone provided by CHDR for the duration of the study period. Patients with internal medical devices such as a pacemaker or deep brain stimulator were excluded from the study, as these could interfere with the ––Withings scale measurements [20]. Participants could not be pregnant or have a severe coexisting illness. Multimedia Appendix 1 illustrates the enrollment pipeline for this study. All patients participated in the trial from the beginning to the end.

### Ethics Approval

This study was approved by the Ethics Committee of BEBO, Assen, the Netherlands (NL69288.056.19) and was registered on ClinicalTrials.gov (NCT04999735). Before any study-related activities, written informed consent was obtained from the patients. Participants received monetary compensation for their time and effort during the trial.

To preserve the privacy of the patients, we deidentified the data and limited the amount of personally identified information collected from the smartphone and the connected devices. The location coordinates of the GPS or the cellular networks were collected as relative coordinates (GPS coordinates with respect to another predetermined location). For the calls and SMS text messaging, only metadata are stored (ie, no actual phone calls or text is being processed and stored). The call and SMS text messaging logs only store a partial phone number, making it impossible to identify the original phone numbers. As for the Withings devices, we created a unique email address (containing patient identifiers) for each patient to couple the Withings device with CHDR MORE, thus eliminating the need for using the patients’ personal email.

### Investigational Technologies

Smartphone and remote sensor data were collected on the CHDR MORE platform. This customizable platform enables the collection, ingestion, and management of data sourced from monitoring devices. The CHDR MORE app was installed on the smartphone of each participant and allows for the unobtrusive collection of smartphone sensor data (sourced from the smartphone’s accelerometer, gyroscope, magnetometer, GPS, light sensor, and microphone) as well as phone usage logs (eg, app usage, battery level, calls, and SMS text messages). The smartphone sensor data provide insights into a participant’s environment, such as location type and travel patterns (GPS), if human voices are present in the environment (microphone), and their physical activity (accelerometer and gyroscope). The phone usage logs give an indication of social activity (through social media and communication apps, calls, and SMS text messages) and smartphone usage (app usage).

The app also collected Withings health data. In this study, 3 Withings devices were used: Withings Steel HR smartwatch (monitors heart rate, sleep states, and a number of steps), Withings Body+ scale (monitors weight and body composition), and Withings BPM Connect (monitors heart rate, systolic blood pressure, and diastolic blood pressure). Together the Withings features reflect the daily physical activities of each of the participants.

This is the first study that aimed to monitor and estimate FSHD symptom severity using smartphone and wearable data. As this was an exploratory longitudinal study, specifically aimed to identify smartphone- and wearable-based features that were predictive of FSHD symptom severity, we did not identify any literature with a similar protocol. To identify these novel features, we decided to collect data from all available sensors and features from the CHDR MORE platform. As the symptoms of FSHD can affect a patient’s travel abilities [21], physical activity, sleep [11,22], and social lives [23], we deemed these features relevant for estimating FSHD symptom severity.

### Data Collection

Participants were monitored for 6 continuous weeks. On days 1 and 42, the clinical evaluations (FSHD Clinical Score and TUG) were performed. On day 1, the CHDR MORE and Withings Health Mate apps were installed on their smartphones. Participants were asked to use their smartphones as normal. Participants were asked to continuously wear their Withings Steel HR smartwatch and weigh themselves and take their blood pressure weekly.

### Data Preprocessing

Before modeling of the data, all sensor data were preprocessed and converted into features using Python (version 3.6.0) and the PySpark (version 3.0.1) library. The raw data were checked for missing values and outliers. Missing values were defined as the absence of data for a specific feature for each day, except for 2 types of measurements: the weekly measurements (eg, weight and blood pressure) and the data related to aperiodic activities (eg, phone calls or SMS text messages). Missing data were not imputed. Outliers were detected by manual visual inspection rather than automated statistical techniques, as our objective was to identify potential outliers that were a result of potential measurement errors rather than participants’ behaviors. Measurement errors were deemed not relevant to our analysis, whereas outliers in behavior could still provide insights into a participant’s symptom severity; therefore, sensitivity analysis was not conducted. Outliers would be subsequently excluded at the discretion of the authors (eg, removing overlapping sleep stages).

### Feature Extraction

All raw data were collected from the smartphone and Withings devices. The features were then aggregated per day, as the symptom severity exhibited on a given day is the focus of FSHD clinical evaluation. As there are no FSHD assessments that assess FSHD symptoms over a longer period, we did not explore other aggregation methods. Discrete features (eg, step count) were summed per day per participant. Continuous features (eg, heart rate) were averaged per day per participant. Table 2 provides an overview of how the features were aggregated based on the data type. Table 3 summarizes which features were extracted from the smartphone and Withings sensors. In addition, Table 3 shows the features that were provided from the MORE platform but were not included for the analysis either due to outliers, missing data, or because they were not of clinical interest.

**Table 2:** A simplified summation of how the features were aggregated based on the data type.

Data type	Time unit	Example feature	Aggregation format	Example aggregation
Count	Per day, per hour	Steps	Sum Mean Maximum	Total steps Max steps per hour Mean steps per hour
Continuous data within a range	Per day	Heart rate	Minimum (5%) Median (50%) Maximum (95%)	Lowest 5% heart rate Median heart rate Maximum 95% heart rate
Duration	Per day	App usage	Total duration Mean duration	Total duration of social apps opened Mean duration of social app use per interaction
GPS coordinates	Per day	Location	Sum Maximum Mean	Total distance traveled Mean and Maximum distance from home

**Table 3:** An overview of the features provided from the MORE platform and the features that were subsequently aggregated per day (with the exception of the body measurements as that was measured once a week).

Category and MORE features		Derived features (per day)	Excluded features
**Demographics**			
	Age		
	Gender		
**Acceleration**			
	Acceleration magnitude	98% acceleration magnitude	Mean acceleration magnitude
	Gyroscope		
	Magnetometer		
**Activity**			
	Steps	Total steps, max steps per hour, and mean steps per hour	
	Heart rate	5%, 50%, and 95% beats per minute (bpm), SD of bpm, and % time spent in the resting state	
	Physical activity duration	Soft, moderate, and intense activity duration	
	Calories		Distance traveled and distance per step
**Apps**			
	App categories: health and fitness, recreational, communication and social, tools, and shopping	Duration, times open	House and home, libraries and demo, reading, and travel
**Body**			
	Diastolic blood pressure, systolic blood pressure, heart pulse (bpm), and weight	Diastolic blood pressure, systolic blood pressure, heart pulse (bpm), and weight	Height (m), fat mass (kg), fat ratio (%), hydration, and muscle mass
**Location**			
	Location categories: commercial, health, home, leisure, public, social, and travel	Total duration at place, total distance traveled, total number of unique places visited, max distance from home, and time spent commuting	
**Social**			
	Calls, voice	Number of calls; number of unique numbers; number of incoming, outgoing, and missing calls; number of calls from known and unknown numbers; total duration of calls; average duration of calls; and % time human voice is detected	SMS text messages
**Sleep**			
	N/A^a^	Number of sleep sessions, total sleep duration, number of sleep phases (awake, light sleep, and deep sleep), duration of sleep phases (awake, light, and deep sleep), time between sleep sessions, and time to fall asleep	

^a^N/A: not applicable.

### Feature Selection

Before modeling, both expert-based manual and automated feature selections were performed. First, features were visually inspected by all authors. Excluded features were based on the number of available data points (eg, 9 participants did not have body composition data) and clinical relevance (eg, time spent on parenting apps was deemed clinically irrelevant). Next, two automated feature selection strategies were compared: (1) stepwise regression and (2) variance inflation factor (VIF). The stepwise regression strategy was an iterative process to select predictive variables that met a significance criterion (*P*<.05). Both forward and backward stepwise regression strategies were used. The VIF was calculated for all pairwise combinations of features to identify collinear features. Pairs of features having a VIF value greater than 10 were identified, and one of the features was subsequently removed for each of the pairs [24]. For comparison, we also fitted the model without any automated feature selection strategies. For each regression model, we applied each of the feature selection strategies.

### Statistical Analysis

Python (version 3.6.0) was used for the data analysis and modeling in conjunction with the Pandas [25], NumPy [26], Matplotlib [27], and Sklearn packages [28]. Three regression models were created: 2 single-task regression models, 1 for each clinical assessment and 1 for each multitask regression model, simultaneously estimating both clinical assessments. For the multitask regression model, a dummy variable was included to denote either the FSHD Clinical Score or TUG.

For all models, linear regression, random forest regressor, and gradient boost regressor were used. A grid search was performed to optimize the hyperparameters for each model. For the Elastic Net linear regression model, we optimized the hyperparameters for the α (range 0-200) and L1 ratio (range 0.0-1.0). For the random forest and gradient boost regressors, we optimized the hyperparameters for the number of estimators (range 0-200), maximum depth (range 1-20), maximum features (range: auto, square root, log2), and maximum leaf nodes (range 2-20). In addition, we optimized the learning rate (range 0.0-1.0) for the gradient boost regressor.

Each model was validated using a group 5 outer-fold and 5 inner-fold nested cross-validation. By using group cross-validation, for each fold, we ensure that the participants in the validation are not also present in the training fold. While the data for all participants were used for the modeling, the cross-validation procedure was used for out-of-sample testing; hence, for each fold of the cross-validation procedure, only a subsample of participants’ data were used. Further, the random forest and gradient boost regressor models only consider a subsample of participants and features per decision tree node. The elastic-net linear regression penalization would also reduce the potential features considered in the model. The cross-validation and models together would improve the generalizability and robustness of the models and therefore reduce the probability of spurious correlations.

We applied each of the feature selection strategies to each of the regression models and compared the results of each model. The model that provided the highest *R*^2^ (variance explained) and the lowest root-mean-square error (RMSE) was selected as the best-performing model. The *R*^2^ and the RMSE explain the variance and the error between the true clinical scores and the predicted scores of the regression models, respectively.

To assess how varying time window affects the model’s estimation of symptom severity, we used an incrementally increasing time window to train the regression models, starting with day 1 and adding the following days until the first 2 weeks of data were included in the training set. To train, optimize, and assess each model’s generalizability, we applied a 5-fold nested cross-validation model. To validate the performance of these models, we used the remaining 4 weeks of data as an external validation data set. To assess the stability of the trained models to yield consistent estimations of symptom severity, we trained the FSHD Clinical Score, TUG, and multitask models on the first week of data. We estimated the symptom severity for the subsequent weeks. We selected the first week, as each patient would have each day of the week represented in their data set.

In sum, we investigated 3 final models, 2 single-task models, and 1 multitask model. For each model, we considered 3 types of regression models (the linear regression, the random forest regressor, and the gradient boost regressor). For each model, we considered 3 feature selection strategies (no automated feature selection, stepwise regression, and VIF); hence, in total, we compared 27 models. Given that we are mainly interested in the comparison of the predictions of single-task and multitask models and the influence of the time windows on the predictions, we reported only the results of these models.

## Results

No patients dropped out of the study. One patient was wheelchair-bound and therefore unable to perform the TUG. The FSHD Clinical Scores ranged between 1 and 13, with a median score of 5. The TUG times ranged between 5.5 seconds and 15.8 seconds, with a median time of 7.7 seconds. Multimedia Appendix 2 illustrates the range of the averaged FSHD Clinical Scores and TUGs.

Before modeling, several features were manually excluded. Nine patients had no body composition (eg, fat and muscle mass) data. As a result, the Withings body composition data (except weight) were excluded from the final analysis. We excluded SMS text message–related features as not all the patients used SMS text messaging (less than 30% of patients), and the SMS text message features were not deemed clinically relevant. Further, we excluded smartphone apps from the analysis that were used by less than 5% of the patients. We did not exclude any outliers as none of the data points were viewed as potential measurement errors. In a previous publication, we provided an overview of the proportion of observations that were missing per feature [15].

The FSHD Clinical Score for 24 participants did not change over the 6 weeks. The scores of the remaining 14 participants changed by +1 or −1 point. The average difference between the day 1 and day 42 TUG scores was 0.38 seconds (95% CI 0.12-0.63). After reviewing the stability of the TUG and FSHD scores, we decided to use the averaged clinical assessment scores as the outcomes for all models. Subsequently, each feature was also averaged over the 6 weeks. These averaged features were used as inputs for the regression models.

**Figure 1:**
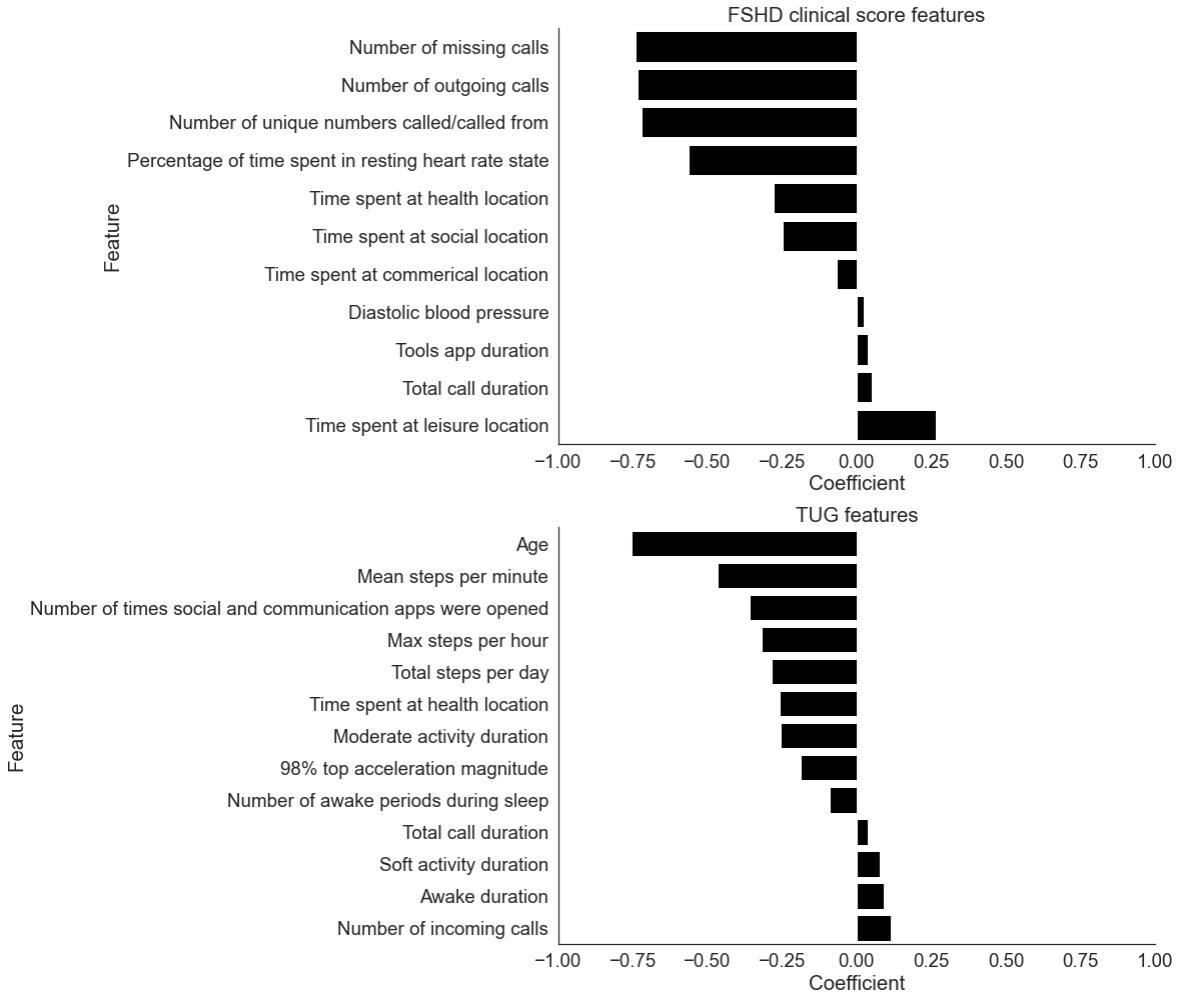
Linear regression coefficients for the features selected by the single-task FSHD Clinical Score and TUG models. Features with a coefficient of zero are not shown. FSHD: facioscapulohumeral muscular dystrophy; TUG: Timed Up and Go.

**Figure 2:**
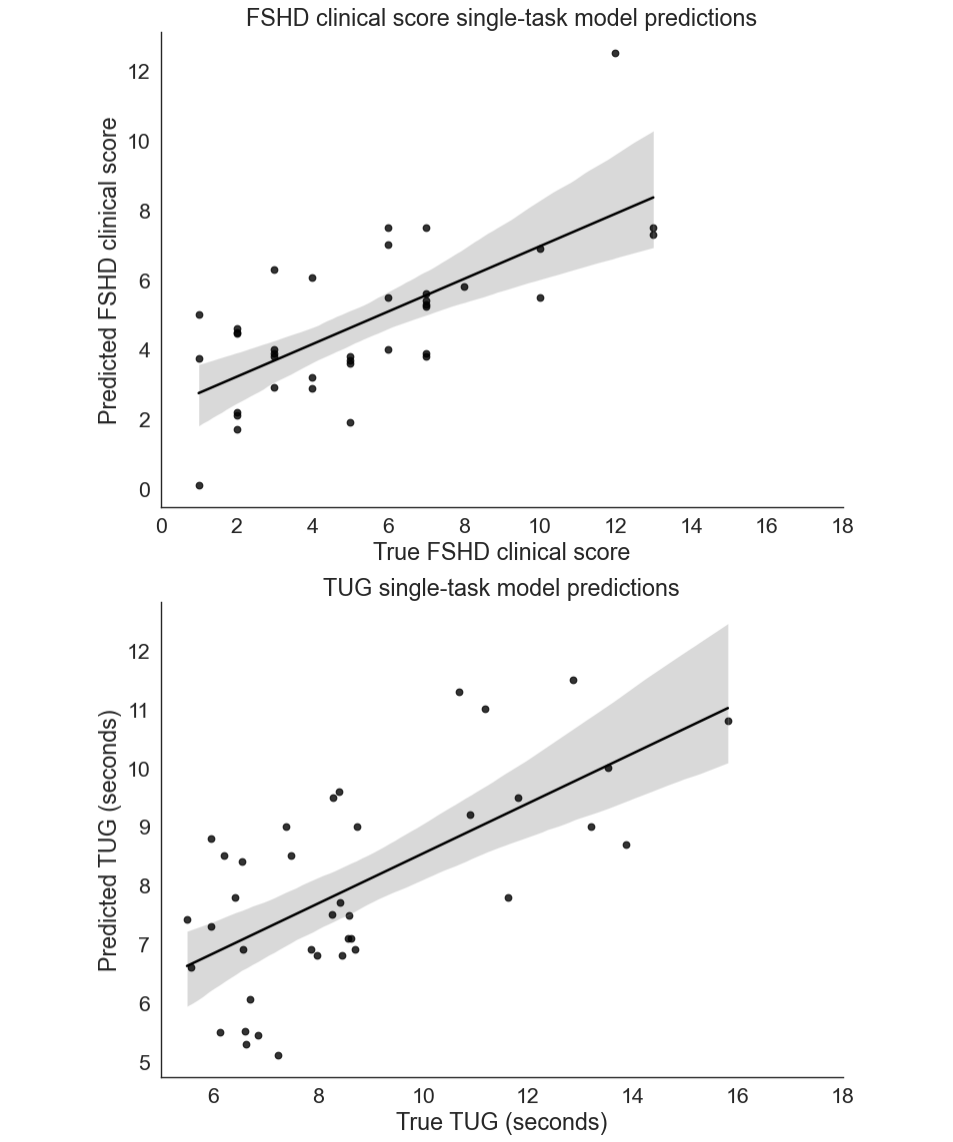
True FSHD Clinical Scores and TUG times against the predicted scores using the respective FSHD Clinical Score and TUG regression models. The lines represent a regression line with a 95% CI band. FSHD: facioscapulohumeral muscular dystrophy; TUG: Timed Up and Go.

Using all 6 weeks of data, we built a single-task model that used the CHDR MORE features to estimate the FSHD Clinical Score for each participant. Comparing the estimated scores and the true FSHD Clinical Score yielded an *R*^2^ of 0.57 and an RMSE of 2.09. This was achieved using VIF-selected features and Elastic Net–penalized linear regression. A total of 11 features were predictive of the FSHD Clinical Score, as seen in Figure 1. The features were related to app usage, blood pressure, location visits, and calling behaviors. Figure 2 (top) shows the estimated FSHD Clinical Score in relation to the actual FSHD Clinical Score.

Similarly, the comparison of the TUG single-task model estimated TUG and the actual TUG yielded an *R*^2^ of 0.59 and an RMSE of 1.66 (seconds) for each participant. This was achieved with forwarding selection stepwise regression and Elastic Net–penalized linear regression. In total, 13 features were predictive of the TUG score (Figure 1). The feature categories related to age, app usage, calling behaviors, sleep, physical activity, and location visits were predictive of TUG. Figure 2 (bottom) illustrates the relationship between the predicted and actual TUG times.

**Figure 3:**
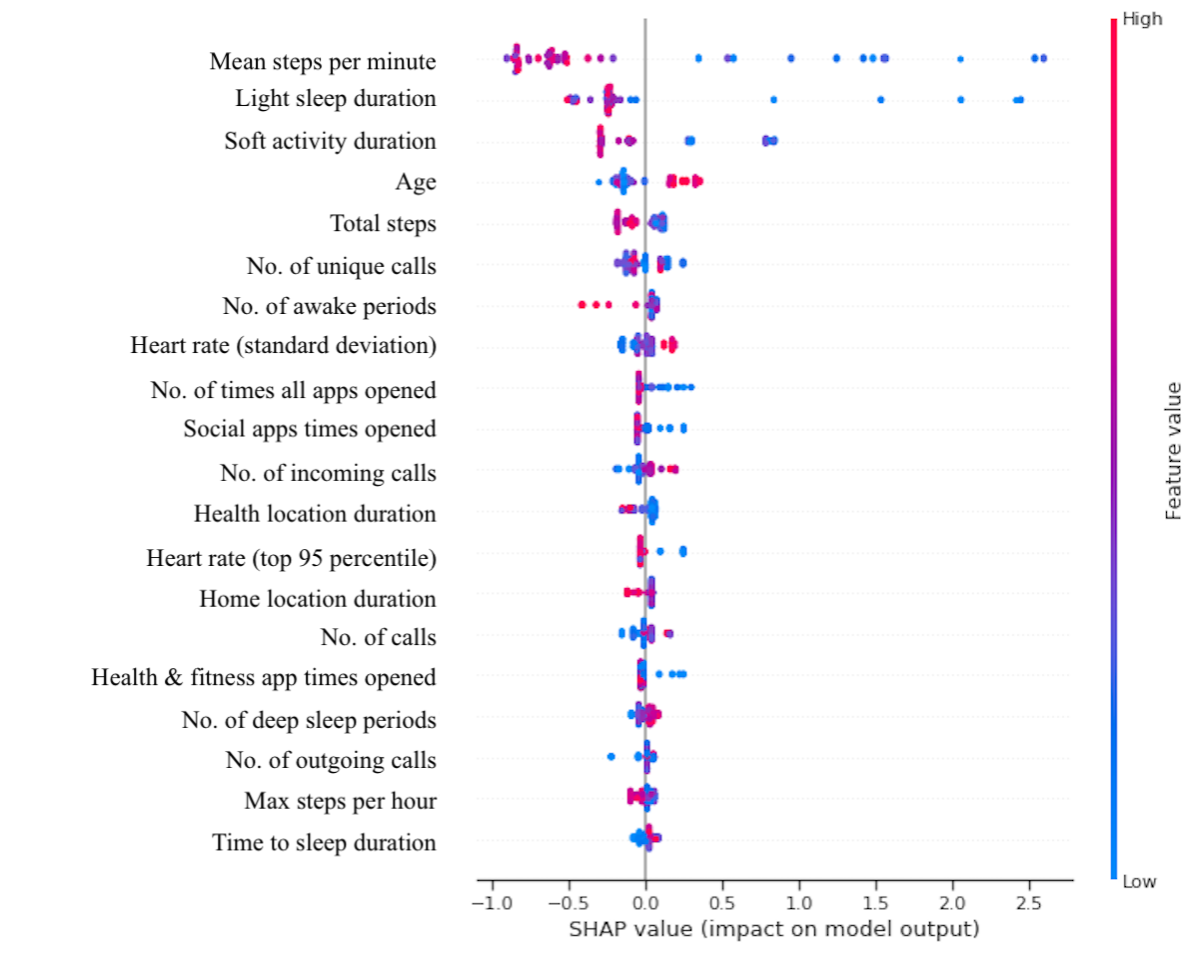
SHAP (SHapley Additive exPlanations) variable importance plot showing the feature importance of the top 20 most important features, in which the features are ranked in descending order. Each scatter point represents one prediction. The color of the scatter point reflects the value of the real data. If the actual value of the data point was high, then the color was red. If the value was low, then the color was blue. The SHAP value, as illustrated on the x-axis, shows the direction and magnitude of each feature’s contribution toward predicting the facioscapulohumeral muscular dystrophy symptom severity.

**Figure 4:**
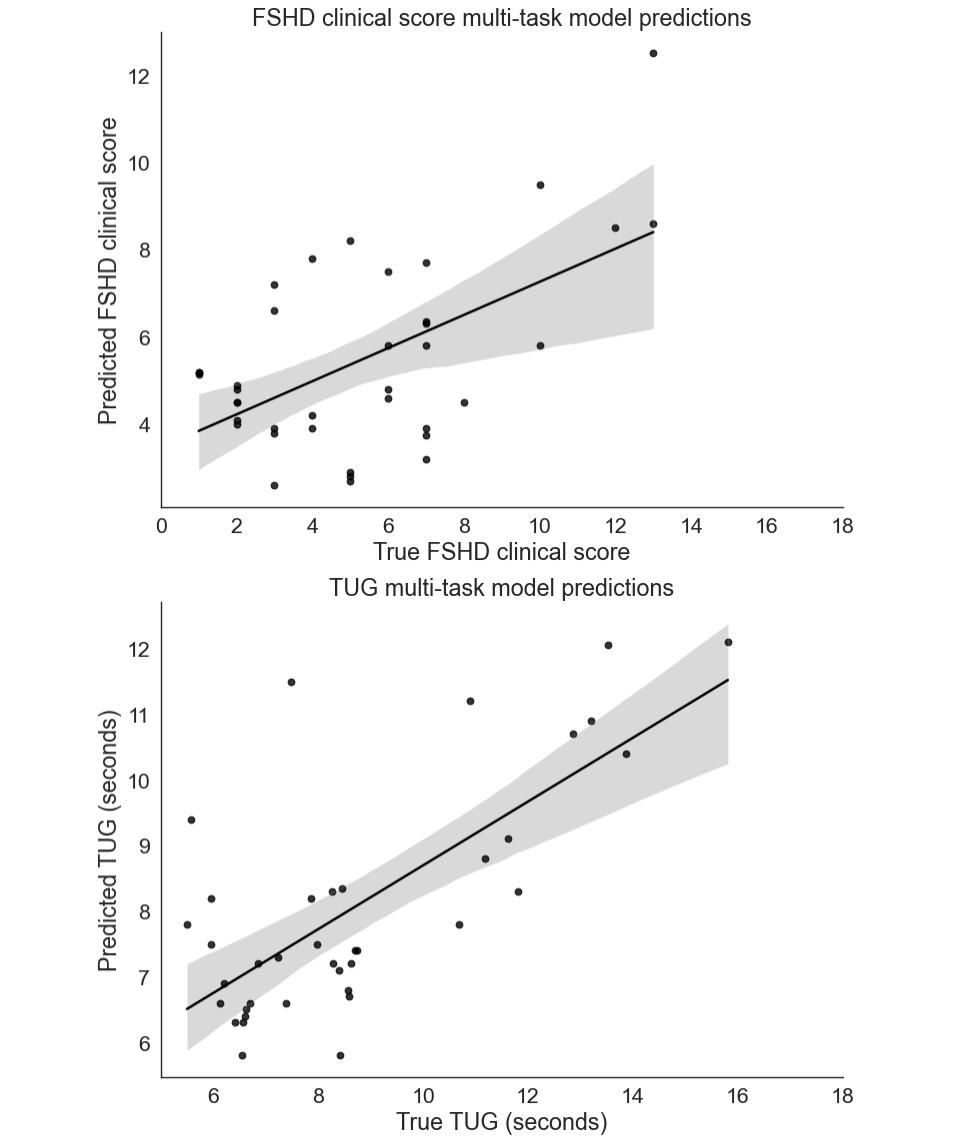
Scatterplot of the estimated FSHD Clinical Scores and TUG times in relation to the actual FSHD Clinical Scores and TUG using the multi-task learning regression model. The lines represent the regression lines with a 95% CI band. FSHD: facioscapulohumeral muscular dystrophy; TUG: Timed Up and Go.

The multitask model achieved an *R*^2^ of 0.74 and an RMSE of 1.89 for the FSHD Clinical Score and TUG prediction together. The same model achieved an *R*^2^ of 0.66 and an RMSE of 1.97 for the FSHD Clinical Score and an *R*^2^ of 0.81 and an RMSE of 1.61 for the TUG separately. The gradient boost regressor selected 50 predictive features. The relative feature importance is presented in Figure 3. The 5 most important features were light sleep duration, total steps per day, mean steps per minute, the number of times the social and communication apps were opened, and the number of incoming calls. Figure 4 illustrates the relationship between the predicted clinical scores and the actual clinical scores.

**Figure 5:**
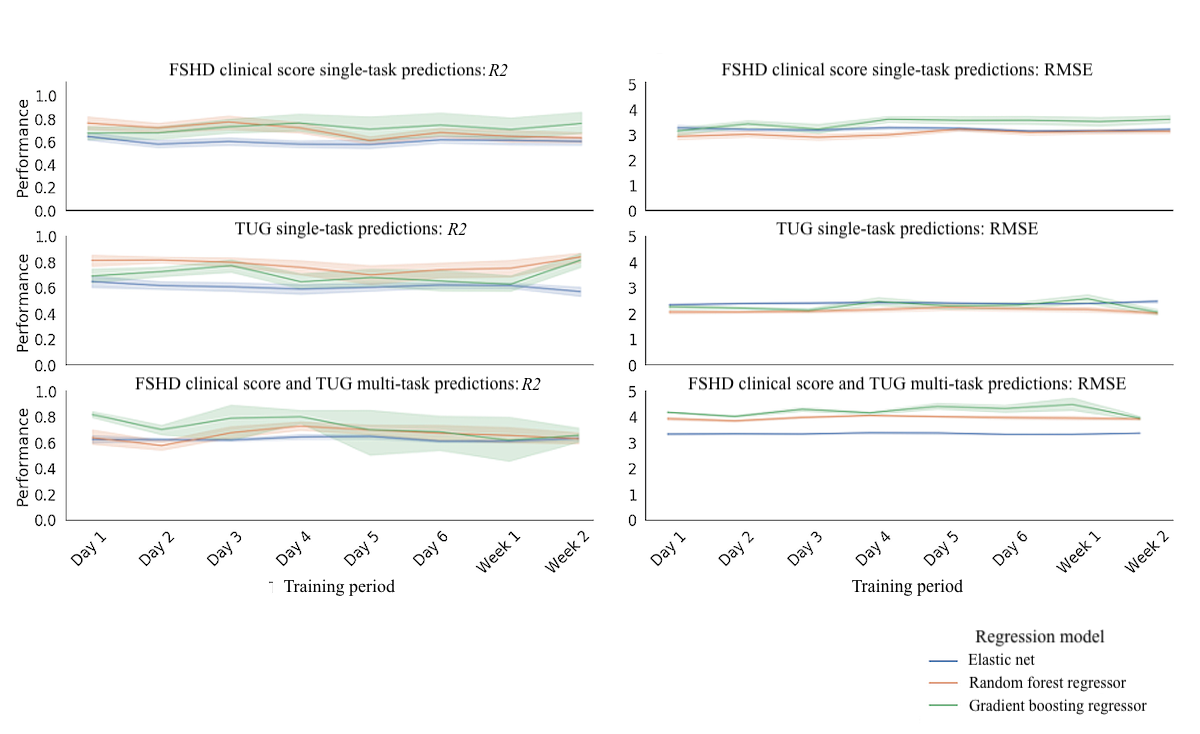
Evaluating the performance of the single-task FSHD Clinical Score, TUG, and the multitask FSHD Clinical Score and TUG regression models trained on an incrementally increasing time window. The colored lines represent the 3 types of regression models trained on the data (Elastic Net, Random Forest Regressor, and Gradient Boosting Regressor). For each model and each incremental time window, the top and bottom plots show the *R*^2^ and RMSE, respectively. The lines represent the median performance, and the bands represent the 95% CI. FSHD: facioscapulohumeral muscular dystrophy; RMSE: root mean square error; TUG: Timed Up and Go.

For each clinical score, we evaluated the effect of different monitoring periods on the estimation of symptom severity. The best performing FSHD Clinical Score single-task model, TUG single-task model, and multitask model yielded the highest *R*^2^ on day 3 (0.70), week 2 (0.86), and day 1 (0.86), and the lowest RMSE on day 3 (2.8), week 2 (1.9), and day 6 (3.4), respectively. As seen in Figure 5, although our analysis has identified windows that yielded the highest *R*^2^ and RMSE, we found that the mean (SD) of the *R*^2^ and RMSE for the FSHD Clinical Score single-task model, TUG single-task model, and multitask model was 0.65 (0.03) and 3.37 (0.19), 0.79 (0.05) and 2.05 (0.09), and 0.76 (0.08) and 4.37 (0.20), respectively.

**Figure 6:**
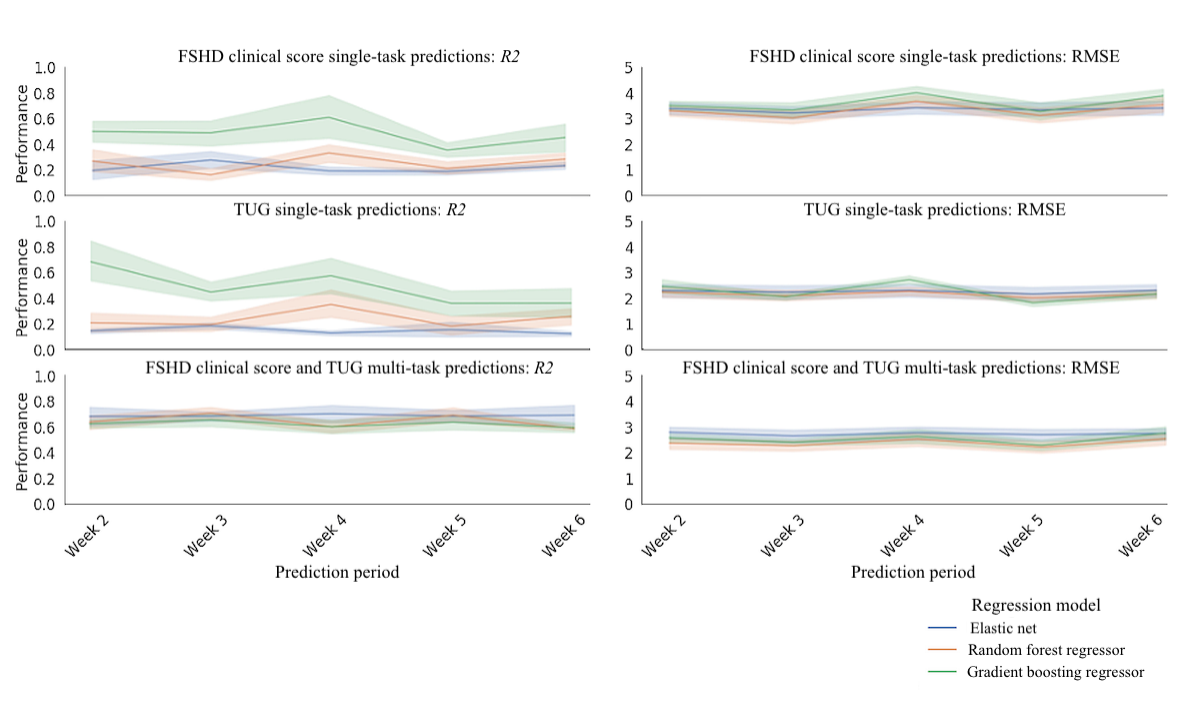
Evaluating the performance of the single-task FSHD Clinical Score, TUG, and the multitask FSHD Clinical Score and TUG regression models trained on the first week of data to estimate symptom severity for the subsequent weeks. The colored lines represent the 3 types of regression models trained on the data (Elastic Net, Random Forest Regressor, and Gradient Boosting Regressor). For each model and each week, the top and bottom plots show the *R*^2^ and RMSE respectively. The lines represent the median performance, and the bands represent the 95% CI. FSHD: facioscapulohumeral muscular dystrophy; RMSE: root mean square error; TUG: Timed Up and Go.

When evaluating the stability, the models trained on a week’s worth of data were used to estimate the symptom severity for subsequent days. We found that the FSHD Clinical Score, TUG, and multitask models achieved median *R*^2^ (median RMSE) of 0.51 (3.66), 0.42 (2.44), and 0.72 (2.61), respectively (as seen in Figure 6).

## Discussion

### Principal Findings

We developed and compared 2 regression models to monitor and estimate FSHD symptom severity outside the clinic with remote sensor data to estimate the FSHD Clinical Score and TUG for each participant. For the first type of model, both clinical assessment scores were separately estimated using 2 single-task regression models. For the second type of model, both clinical assessment scores were simultaneously estimated using a multitask regression model.

The 2 single-task models selected features that were uniquely predictive of each of the clinical scores. In addition, the models’ selected features were found to be predictive for both scores (time spent at health locations and total call duration). Other studies have found that (a modified version of) the TUG significantly correlated to the FSHD Clinical Score [12,29], indicating that these clinical scores share mutual information. Simultaneously estimating multiple tasks with shared features can improve the model performance [30-32]. This supports the notion that a multitask approach would improve the estimation of FSHD symptom severity.

Indeed, the multitask modeling of both the FSHD Clinical Score and the TUG outperformed the single-task models. Additionally, the multitask model identified features not selected as important by the single-task models (eg, sleep and the resting heart rate). The clinical assessments and their respective single-task models only captured a limited range of disease symptoms, which misses the opportunity to model other aspects of the disease (eg, sleep impairments [33,34] and arrhythmic abnormalities [35]). The multitask model, however, identified features representative of a broader range of FSHD symptoms. As shown in the SHAP (SHapley Additive exPlanations) plot (Figure 3), participants with a higher mean step per minute, light sleep duration, soft activity duration, and total steps (indicated by the red feature value) had lower SHAP values. This indicates that participants with more physical activity and better sleep quality had a lower FSHD Clinical Score and TUG. Although the multitask model outperformed the single-task models, the multitask model required approximately twice as many features as the single-task models. Using fewer features could be considered beneficial as it reduces the number of sensors needed. Additionally, it eases the interpretation of the results. Therefore, there is a tradeoff between the performance of estimation of disease severity and the complexity of the data set and model. However, given that the multitask model showed an important improvement over the single-task models, we recommend using the multitask model for future estimation of the FSHD Clinical Score and TUG.

It is critical to determine how much data are needed to obtain reliable inferences without burdening the patients and the clinicians. Insufficient data can lead to inaccurate extrapolations, whereas excessive data can lead to wasted time and resources. This study investigated how long a patient needs to be monitored to estimate symptom severity reliably. Our results demonstrated that behaviors exhibited that based on our sample, the optimal time window (based on the highest *R*^2^ and lowest RMSE) varied for each task. The multitask model yielded the overall highest *R*^2^ based on a training data set of the first day. Although we identified that 5 days of data seem sufficient for training the multitask model, a longer or shorter time window would still provide consistent estimation of the symptom severity. However, our results also demonstrate that selecting any time window between days 1 and 14 would produce relatively stable results. Our results also demonstrated that training the multitask model on the first week of data allowed for constant and reliable estimations of symptom severity for the subsequent weeks. This further supports the notion that the multitask should be used to estimate the clinical scores for longitudinal studies.

The agreement between the clinical scores and the remotely monitored features did not achieve 100% adherence. This may be due to the sensors being unable to capture specific aspects of the clinical score. For example, features captured by the remote monitoring system may not provide sufficient proxies for arm, scapular, and abdominal weaknesses (which the FSHD Clinical Score specifically addresses). Adding additional sensors and features could potentially allow for more complete modeling of FSHD. For example, an additional accelerometer could try to capture arm swings [36] or detect the (limited) shoulder range of motion [37]. Another explanation for the imperfect model fit is that the clinical scores have limited accuracy in capturing disease severity. There can be variation within a specific clinical score, as patients with the same scores may exhibit different FSHD symptoms. For example, patients with scores between 2 and 4 may have impairments related to facial muscles and upper limbs, whereas others may be unable to walk on their heels [11].

The clinical scores provide snapshots of muscular strength and function, whereas the remote monitoring approach provides a more continuous measure of (FSHD-related) social and physical activity. Additionally, the clinical scores were assessed at the clinic, whereas the sampling of the remotely monitored features occurred at home, and in daily practice. Altogether, these 2 clinical scores may not be the optimal clinical assessment strategies for fully assessing FSHD symptom severity. These are only 2 of several FSHD-related assessments that can be used in a clinical trial. The remotely monitored features may show different correlations with other FSHD-related assessments such as the Clinical Severity Scale for FSHD [38,39] and the Pittsburgh Sleep Quality Index [39,40]. Although the remotely monitored features may not correlate strongly with the 2 clinical scores, they still provide relevant insights into FSHD-related symptoms. Our multitask model could prove to be a promising tool for monitoring the FHSD severity based on patients’ everyday activities outside the clinic.

Although the models cannot replace the TUG or FSHD Clinical Scores for estimating the disease severity, these models can potentially be used as a (complimentary) tool in clinical studies. When validated in longitudinal studies, given the continuous sampling of data from multiple sensors, this FSHD tool could potentially be used to track the symptom severity for long periods of time without patients having to visit a clinic. Previous studies have demonstrated that this approach of using smartphone-based models to quantify medication responses can be advantageous [37,38]. When implemented in a clinical trial, the FSHD tool might be evaluated as a tool to monitor drug effectiveness by tracking drug-induced changes in FSHD symptom severity [41]. Additionally, it might enable the identification of improvements in specific aspects of the disease severity (eg, muscle function or sleep quality). Therefore, remote monitoring might aid clinicians’ assessments of a patient’s status during a clinical trial based on the review of the patient’s in-clinic assessments and out-of-clinic daily activity.

We present an FSHD tool that estimates the FSHD Clinical Score and TUG using smartphone and remote sensor data. The conclusions drawn from this study are preliminary in view of the relatively small sample size and cross-sectional study nature. Given the short observation period, we did not expect changes in the patients’ FSHD scores. As a result, we could not validate the use of the model to estimate changes in the FSHD severity over time. A trial where the FSHD clinical score is expected to change could help validate the FSHD tool’s capacity to detect changes in FSHD symptom severity. Additionally, the FSHD tool could be improved by including more patients with FSHD and adding other remote sensors. All in all, the remote monitoring approach presented here could be a promising tool for monitoring FSHD severity outside the clinic environment.

### Conclusions

We presented a smartphone-based and remote sensor–based FSHD tool that can estimate a patient’s FSHD symptom severity. This is the first study to demonstrate how to monitor patients with FSHD remotely and subsequently model their FSHD Clinical Score and TUG simultaneously. The tool holds potential for monitoring disease progression and drug intervention effects outside the clinic, pending a longitudinal follow-up study to validate the capacity of the FSHD tool to detect changes in the disease severity score over time due to disease progression or drug intervention.

## Appendix

Multimedia Appendix 1 Pipeline of the enrollment process for this study. 41 FSHD patients were recruited, 2 patients were excluded, and 1 patient refused.

Multimedia Appendix 2 Distribution of Averaged FSHD Clinical Score (between day 0 and day 42 of the study). Distribution of Averaged TUG times (seconds) (between day 0 and day 42 of the study).

## Abbreviations

CHDR: Centre for Human Drug Research
FSHD: facioscapulohumeral muscular dystrophy
RMSE: root-mean-square error
SHAP: SHapley Additive exPlanations
TUG: Timed Up and Go
VIF: variance inflation factor

## Data Availability

The data acquired during this study are available from Centre for Human Drug Research. Given that the data are under license for the current study, they are not publicly available. The data are, however, available on permissions from Facio Therapies.
