# Supplementary figures and images for: Smartphone and Wearable Sensors for the Estimation of Facioscapulohumeral Muscular Dystrophy Disease Severity: Cross-sectional Study
Source: JMIR Form Res. 2023 Mar 15;7:e41178. doi: 10.2196/41178 (PMC10131943; doi:10.2196/41178)

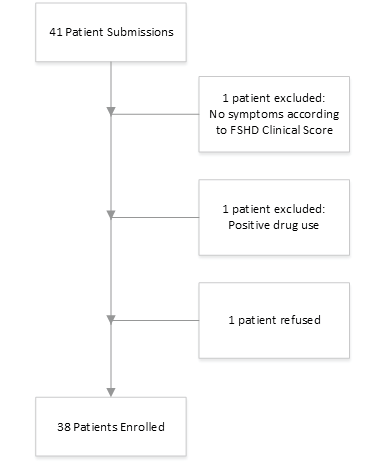

Supplement: Multimedia Appendix 1 [file formative_v7i1e41178_app1.png]

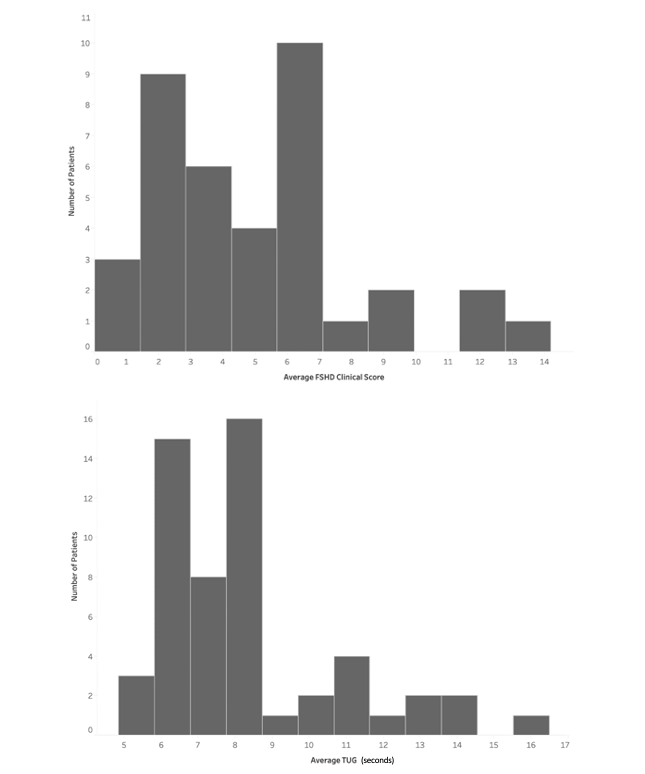

Supplement: Multimedia Appendix 2 [file formative_v7i1e41178_app2.png]
